# Limbal fibroblast conditioned media: A non-invasive treatment for limbal stem cell deficiency

**Published:** 2011-03-08

**Authors:** H. Amirjamshidi, B.Y. Milani, H.M. Sagha, A. Movahedan, M.A. Shafiq, R.M. Lavker, B.Y.T. Yue, A.R. Djalilian

**Affiliations:** 1 Department of Ophthalmology and Visual Sciences, University of Illinois at Chicago, Chicago, IL; 2 Department of Dermatology, Northwestern University Feinberg School of Medicine, Chicago, IL

## Abstract

**Purpose:**

Limbal fibroblasts are known to regulate the maintenance and differentiation of the corneal epithelium including the limbal epithelial stem cells. This study examined the effect of limbal fibroblast conditioned media in a mouse model of limbal stem cell deficiency.

**Methods:**

Limbal stem cell deficiency was created in C57/Bl6 mice by performing a limbus to limbus epithelial debridement. The mice were treated topically for 3 weeks with conditioned media derived from human limbal fibroblasts. The control mice were treated with skin fibroblast conditioned media or Dulbecco’s serum-free medium.

**Results:**

The mice treated with limbal fibroblast conditioned media demonstrated substantial growth of corneal type epithelial cells on the corneal surface with less conjunctival goblet cells. By contrast, the control treated corneas were found to be covered primarily by conjunctival type epithelium.

**Conclusions:**

Cell culture media conditioned by limbal fibroblasts appear to contain factor(s) that are therapeutically beneficial in a model of limbal stem cell deficiency. The current results further support the notion that the essential limbal stem cell niche is provided by limbal fibroblasts and suggest a new, non-invasive option in the treatment of limbal stem cell deficiency.

## Introduction

The corneal epithelium, like many other epithelial surfaces, undergoes constant renewal by a population of local adult stem cells. The corneal epithelial stem cells are located primarily in the basal aspect of the limbal epithelium, hence they are known as the limbal stem cells [1,2]. Under various pathologic conditions, such as severe immunologic disorders or chemical injuries, the limbal stem cells may be damaged or destroyed [3]. In such situations, the corneal epithelium is gradually replaced by conjunctival epithelium which has significant implications. In particular, patients with limbal stem cell deficiency, develop non-healing epithelial defects, neovascularization, and scarring of the cornea resulting in profound loss of vision [3]. Currently, most treatments for limbal stem cell deficiency involve surgical replacement of the limbal stem cells from a healthy donor eye [4]. There are very few non-surgical treatment options available especially for patients who have subtotal limbal deficiency or those with localized dysfunction of the limbus. Most non-surgical treatments such as anti-inflammatory therapy, autologous serum tears, or scleral contact lenses are aimed at restoring a healthy ocular surface and environment for the limbal stem cells [5-7].

Recent studies have highlighted the critical role of the stromal microenvironment (aka niche) in the maintenance and homeostasis of the corneal and limbal epithelium [8-11]. While the specific features of the limbal niche have not been fully charaterized, it likely includes both cellular and extracelular factors [8-12]. The stromal fibroblasts, in particular, appear to be one of the important components of the limbal niche, given their intimate interactions with the epithelium through the production of cytokines and growth factors [11,13,14]. Previous studies have demonstrated the essential role of the corneal and limbal fibroblasts in regulating epithelial proliferation, differentiation, and wound healing [13-19]. Likewise, limbal fibroblasts appear to play an important role in maintaining the limbal epithelial phenotype [12,20,21].

In this study, we sought to determine whether conditioned media derived from cultured limbal fibroblasts would have a therapeutic effect in an in vivo model of limbal stem cell deficiency.

## Methods

### Limbal stem cell deficiency model

**Figure 1:**
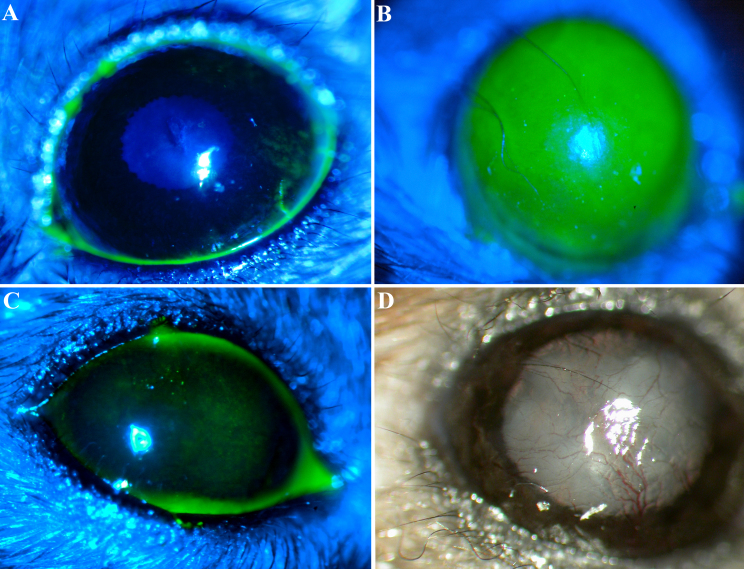
Mouse model of limbal stem cell deficiency created using limbus to limbus scraping. Fluorescein staining pattern of a normal cornea showing minimal staining due to dryness (**A**). Immediately after total corneal epithelial debridement there was a total epithelial defect (**B**). Three weeks after total removal of the corneal epithelium, fluorescein staining revealed diffuse punctate epithelial staining (**C**). Two months after total debridement, the cornea has developed progressive superficial neovascularization (**D**).

A mouse model of limbal stem cell deficiency was developed in part based on the model described by Pal-Ghosh et al. [22]. The model was slightly modified such that instead of a 2.5 mm diameter scraping of the corneal epithelium, the entire corneal epithelium from limbus to limbus was scraped using a blunt spatula. Although the original model described by Pal-Ghosh et al. [22] was not intended to induce limbal stem cell deficiency, other investigators have shown that limbus to limbus epithelial debridement results in limbal stem cell deficiency in mice [20]. The procedure was performed in 3 to 6-month-old C57Bl/6 mice that were anesthetized with an intraperitoneal injection of ketamine (100 mg/kg) and xylazine (5 mg/kg) followed by several drops of topical 0.5% proparacaine. Care was taken not to injure the corneal stroma. At the conclusion of the procedure, fluorescein staining was used to confirm complete removal of the corneal epithelium (Figure 1A,B). All the procedures complied with the ARVO Statement for the Use of Animals in Ophthalmic and Vision Research.

To establish the feasibility of this model, mice that had undergone limbus to limbus debridement were euthanized at various time points from 2 weeks to 2 months and subjected to immunostaining.

### Cell culture and conditioned media

Limbal fibroblast cultures were developed from human cadaver corneas. Human cadaver corneas stored in Optisol for less than 2 weeks were obtained from the Illinois Eyebank (Chicago, IL). After removing and discarding the central corneas with an 8 mm trephine, the remaining limbal rings were incubated for 2 h at 37 °C in 2 mg/ml Dispase II (Invitrogen, Carlsbad, CA) in phosphate buffered saline (PBS).The corneal epithelium and the Descemet’s membrane were then separated and the remaining limbal stroma was cut into 1 mm×1 mm pieces and incubated at 37 °C in 0.1% collagenase (Sigma, St. Louis, MO) in Dulbecco’s minimal essential medium (DMEM)/F12 for 1.5 h on a shaker. The isolated keratocytes were expanded and differentiated into fibroblasts by culturing in DMEM supplemented with 10% fetal bovine serum. After 3–4 passages, when the cells had reached at least 80%–90% confluence, the media were changed to DMEM without serum. Twenty four hours later the media were collected and used immediately.

As a control for limbal fibroblasts, skin fibroblast cultures were initiated from fresh human foreskin tissue. After washing the tissue in PBS and 5% betadine for 5 min, the tissue was cut into 5×5 mm pieces and incubated overnight at 4 °C in 2 mg/ml Dispase II in PBS . After removing the epidermis, the dermis was cut into smaller pieces and used as explants to initiate dermal fibroblast cultures in DMEM supplemented with 10% fetal bovine serum. After 3–4 passages, when the cells had reached at least 80%–90% confluence, the medium was changed to DMEM (without serum). The media was collected 24 h later.

### Conditioned media treatment

Mice were treated immediately after limbus to limbus epithelial scraping. They were treated topically with limbal fibroblast conditioned media, skin fibroblast conditioned media, or DMEM. The treatment was done once daily by placing the mouse under anesthesia and applying the media (20–30 µl) on the cornea for 2 h. This was accomplished by applying the media with a micropipette such that it formed a droplet over the entire cornea. The mice were monitored every 15 min and as the drop evaporated additional media was applied to maintain a droplet over the cornea throughout the 2 h that the mouse was kept under anesthesia. For the first week, all of the mice also received topical bacitracin ointment to minimize the risk of infection. Treatment was scheduled to continue for 3 weeks in all the mice however periodically some of the mice died under anesthesia (presumably due to the stress of daily anesthesia) and therefore to maintain adequate sample size, the mice that were treated for a minimum of 2 weeks were included in the study. At the end of the 3 week period, the remaining mice were euthanized and examined by immunostaining. A total of 10 mice were treated with the limbal fibroblast conditioned media while another 10 control mice received either skin fibroblast conditioned media (6 mice) or DMEM (4 mice).

### Immunostaining

To assess the entire corneal epithelium, whole mount staining as described by Pal-Ghosh et al. [22] was performed. Eyes were enucleated and fixed for 2 h with pre-chilled 4:1 dilution of 100% methanol and DMSO at −20 °C and then stored in 100% methanol. Using the dissecting microscope, the retina, lens, and iris were removed. Corneas were transferred into Tris-buffered saline (TBS) in a graded methanol series (70%, 50%, and 30% methanol and TBS, 30 min each). All incubations were performed with gentle shaking at room temperature, unless otherwise specified. The corneas were washed in TBS and 0.03% Triton X-100 twice, for 20 min each, and were incubated at room temperature for 1 h with blocking buffer (1% BSA and donkey serum in 1× TBS). They were then incubated overnight at 4 °C with primary antibody rat monoclonal anti-keratin 8 (K8) antibody (1:40, monoclonal antibody TROMA-I developed by Philippe Brulet and Rolf Kemler obtained from the Developmental Studies Hybridoma Bank developed under the auspices of the NICHD and maintained by The University of Iowa, Department of Biology, Iowa City, IA) or goat polyclonal anti-keratin 12 (K12) antibody (1:50; Santa Cruz Biotechnology, Santa Cruz, CA). The corneas were further washed three times with TBS and 0.03% Triton X-100 for 30 min each and were then incubated overnight at 4 °C with donkey anti-rat or donkey anti-goat secondary antibody, followed by additional washing with TBS and 0.03% Triton X-100 three times for 30 min each. To achieve the best result in flattening the corneas, four incisions were made in each cornea and the specimen was placed epithelial side up on a slide. The slides were counterstained with DAPI, visualized using a Zeiss Axiovert fluorescence microscope, and photographed with an AxioCam (Carl Zeiss, Thornwood, NY) camera.

In addition to whole mount staining, cross sections were also obtained. Mouse whole eyes were embedded in OCT and then cut into 8 μm sections. The frozen sections were fixed for 10 min in chilled acetone and blocked at room temperature with 10% donkey serum for 1 h. The sections were incubated overnight at 4 °C with rat monoclonal anti-K8 or goat polyclonal anti-K12. For negative controls, the sections were incubated in either isotype control or without the primary antibody. A fluorescein-conjugated goat anti-rat or rhodamine conjugated rabbit anti-goat was used as the secondary antibody for 1 h at room temperature followed by counterstaining with DAPI. The sections were visualized using a Zeiss Axiovert fluorescence microscope, and photographed with an AxioCam camera.

### Quantification and statistical analysis

The expression of K8 and K12 in the cornea by wholemount staining was quantified using the Metamorph software (Molecular Devices, Sunnyvale, CA). For each image, the number of pixels for each color (green or red) was divided by the total pixels to determine the percentage. The mean percentages for K8 and K12 in the three treatment groups were compared using ANOVA.

## Results

### Conjunctivalization after total corneal epithelial removal

**Figure 2:**
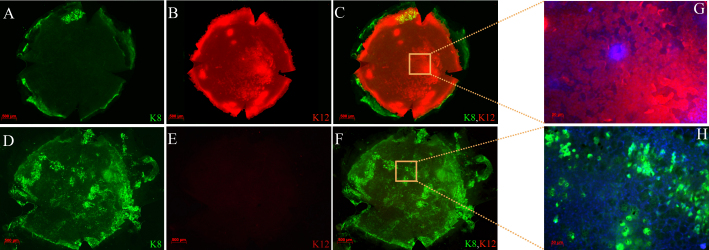
Expression of keratin 8 (K8) and keratin 12 (K12) in normal and limbal deficient mouse corneas. Normal (**A**-**C**) and limbal stem cell deficient corneas after limbus to limbus epithelial debridement (**D**-**F**). Wholemount staining shows the absence of goblet cell marker K8 (green, **A**), and the presence of K12 (red, **B**) in a normal cornea while in limbal deficient corneas there was the presence of K8 (green, **D**) and absence of K12 (red, **E**). An overlay of the K8 and K12 staining is shown for normal (**C**) and limbal deficient (**F**) corneas. High magnification of the double staining along with DAPI staining (blue) is shown for normal (**G**) and limbal deficient (**H**) corneas.

**Figure 3:**
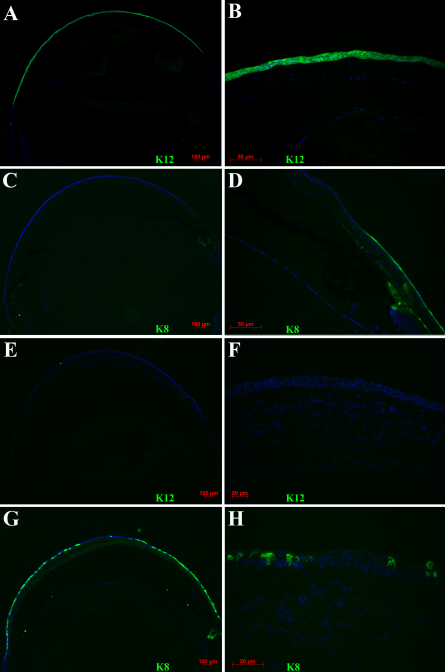
Expression of keratin 8 (K8) and keratin 12 (K12) in normal and limbal deficient mouse corneas. Cross sections of normal (**A**-**D**) and limbal stem cell deficient corneas after limbus to limbus epithelial debridement (**E**-**H**). Normal corneal epithelium expressed K12 (**A**, **B**) but no K8 staining (**C**). Staining for K8 is evident only in the conjunctiva and limbal area (**D**). By contrast, limbal deficient corneas demonstrated no K12 (**E**, **F**) but abundant K8 (**G**, **H**) staining. Nuclei are stained with DAPI in blue.

To assess the effect of limbus to limbus epithelial debridement as a model of limbal stem cell deficiency, mice corneas were examined at various time points ranging from 2 weeks to 2 months (without applying any specific treatment). Fluoresecin staining revealed diffuse punctate epithelial staining from limbus to limbus consistent with a clinical pattern of conjunctivalization due to limbal deficiency (Figure 1C). In addition, most mice developed fine superficial neovascularization starting in the second week. The neovascularization was prominent particularly in the periphery however if left untreated over time it would progress to involve the central cornea (Figure 1D). Further confirmation of limbal deficiency was done by demonstrating the presence of goblet cells by wholemount staining and immunostaining of corneal cross sections for K8, a maker of conjunctival goblet cells (Figure 2 and Figure 3). In contrast to normal corneas, limbal deficient corneas did not express any corneal specific K12 (Figure 2 and Figure 3).

### Effect of limbal fibroblast conditioned media on the development of limbal stem cell deficiency

**Figure 4:**
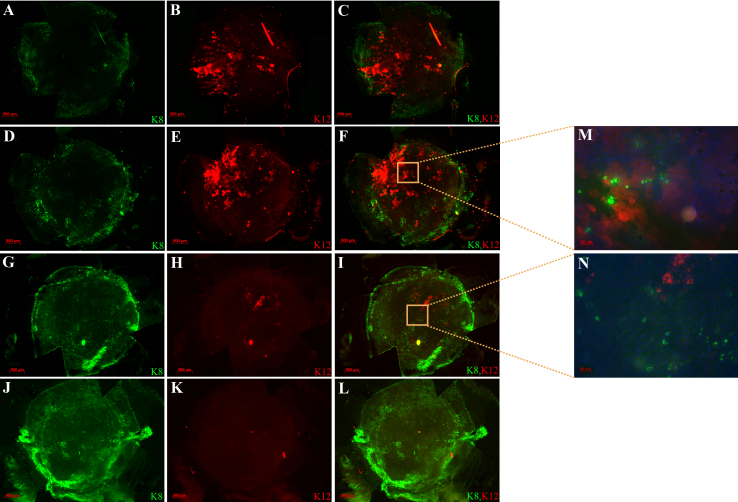
Whole mount staining of mouse corneas after limbus to limbus scraping followed by 3 weeks of treatment. Corneas treated with limbal fibroblast conditioned media had much fewer K8 positive goblet cells in the central cornea (green **A**, **C**, **D**, **F**) but showed consistent presence of K12 positive corneal epithelial cells (red, **B**, **C**, **E**, **F**). Corneas treated with DMEM (**G**-**I**) and skin conditioned media (**J**-L) demonstrated more abundant K8 positive cells (green, **G**, **I**, **J**, **L**) and minimal to no K12 staining (red, **H**, **I**, **K**, **L**). A magnified view with DAPI is shown in **M** and **N**.

**Figure 5:**
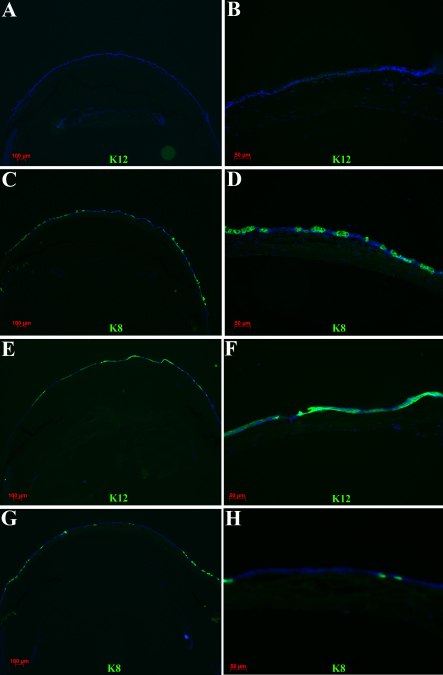
Expression of corneal differentiation marker in mouse corneas after limbus to limbus epithelial scraping and treatment with conditioned media. Corneas treated with skin conditioned media showed no K12 (**A**, **B**) with abundant expression of K8 (**C**, **D**). Eyes treated with limbal fibroblast conditioned media demonstrated K12 staining (**E**, **F**) and reduced K8 positive cells (**G**, **H**).

Mouse eyes were treated with the various conditioned media for 2–3 weeks following limbus to limbus scraping of the epithelium. Clinical examination of the corneas treated with limbal fibroblast conditioned media demonstrated waves of normal staining epithelium (no punctuate staining) arising from the limbus. On wholemount staining, these eyes demonstrated the presence of K12 in the cornea and substantially less K8 compared to skin conditioned media and DMEM (Figure 4). In contrast, the corneas treated with DMEM or skin fibroblast conditioned media demonstrated rare clonal growth of K12 positive cells (Figure 4). A similar expression pattern was seen on cross sections (Figure 5).

**Figure 6:**
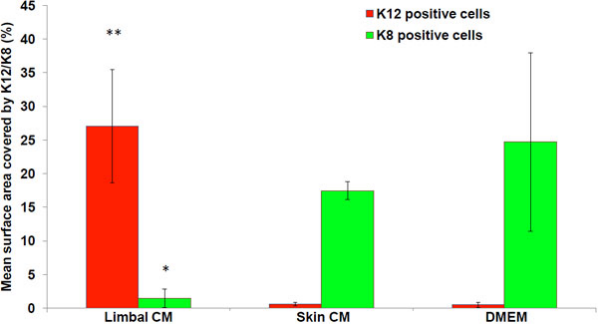
Percentage of the surface area covered by K12 and K8 positive cells. The mean surface area covered by K12 positive cells (red bars) was significantly higher in limbal fibroblast conditioned media compared to the skin conditioned media and DMEM groups (**p<0.001). Similarly, the mean surface area covered by K8 positive cells (green bars) was significantly lower in the limbal conditioned media group compared to the skin conditioned media and the DMEM groups (*p<0.01). The error bars represent the standard deviation.

Quantification of the treatment effects was performed by analyzing the wholemount images using the Metamorph software. The mean surface area covered by K12 positive cells in the limbal conditioned media group was 27.0±8.4% compared to 0.59±0.27% in the skin conditioned media and 0.50±0.39% in the DMEM (p<0.001). Similarly, the mean surface area covered by K8 positive cells was 1.48±1.38 in the limbal conditioned media group compared to 17.47±1.34 in the skin conditioned media and 24.7±13.3 in the DMEM (p<0.01; Figure 6).

## Discussion

In this study we have demonstrated that cell culture media conditioned with human limbal fibroblasts can have a therapeutic benefit in a mouse model of limbal stem cell deficiency. In particular, topical therapy with the conditioned media appears to promote a corneal epithelial phenotype with a concomitant decrease in conjunctival type epithelium on the corneal surface. These results can potentially be explained by two different mechanisms. The first is that factors secreted by the limbal fibroblasts stimulate the proliferation and differentiation of the remaining viable limbal stem cells, while another explanation is that it induces trans-differentiation of other stem cells on the ocular surface. Based on the model used in this study, the former may be the more likely explanation for our findings, namely, that a limbal fibroblast conditioned media specifically stimulates the remaining limbal stem cells to proliferate and differentiate into corneal epithelium.

The model used in this study was partly based on the model described by Pal-Ghosh et al. [22] with a slight modification such that instead of a 2.5 mm epithelial scraping, the entire corneal epithelium from limbus to limbus was removed. Similar to what they have described, following scraping, the cornea was covered by conjunctival type epithelium as evidenced by the expression of K8 positive goblet cells and the absence of keratin 12 expressing corneal epithelial cells. One other difference from their model is that the limbus to limbus debridement also often leads to fine superficial neovascularization especially in the periphery. We have anecdotally observed that in cases where the scraping was done more traumatically, such that the eye developed more inflammation, the cornea in turn developed more severe vascularization. Limbus to limbus epithelial debridement using an Algerbrush II has likewise been shown to cause limbal stem cell deficiency and superficial neovascularization [20].

The main reason why the current model was chosen over the more traditional chemical injury models is that it does not typically induce severe inflammation and destruction of the deeper layers of the cornea. This allows for a better isolation of the treatment effect on the epithelium. In the chemical injury models, given the significant inflammation, neovascularization, and scarring responses, it is much more difficult to determine the mechanism by which a particular treatment affects the outcome. Another advantage of our model is that it is more analogous to clinical cases of partial limbal deficiency since there is likely to be some remaining limbal stem cells after limbus to limbus epithelial debridement. Previous rabbit studies have shown that simple mechanical scraping of the cornea and the limbus in rabbits leads to incomplete removal of limbal stem cells [23]. Although mice do not seem to have deep limbal crypts, as in humans, where limbal stem cells could be protected from simple mechanical scraping, limbal stem cells could still survive in the limbal conjunctiva [24]. A limitation of our model and this study, however, is that the remaining limbal stem cells in each mouse could not be determined before starting treatment. In other words, following limbus to limbus epithelial debridement, each mouse may have a different number of surviving limbal stem cells which in turn will affect their final outcome.

An interesting question that comes up regarding this model, as well as clinical cases of partial limbal stem cell deficiency, is that if there are remaining viable limbal stem cells, why then does the cornea become covered by conjunctival epithelium. One likely explanation may be that the limbal niche is disturbed in such cases. It is conceivable that once the barrier effect provided by the limbus is compromised and the conjunctiva begins to grow over the cornea, the remaining limbal stem cells are no longer supported by a suitable environment and thus are unable to proliferate and differentiate properly. Limbal fibroblast conditioned media, as shown in this study, may provide some of the factors necessary to help stimulate the limbal stem cells to begin supplying corneal epithelial cells again. The restoration of normal limbal function without transplanting limbal stem cells, is consistent with other clinical therapies which have been shown to reverse limbal stem cell deficiency by restoring a healthy ocular surface or environment for the limbal stem cells [5-7,25].

Previous studies have clearly shown the importance of corneal and limbal fibroblasts in corneal epithelial growth and differentiation [13-20]. While the critical factors produced by corneal or limbal fibroblasts have not been fully characterized, based on earlier work, it may include several cytokines such as hepatocyte growth factor, keratinocyte growth factor, and IL-6 [11,13-15]. In addition, it may include corneal specific factors that are crucial for the corneal epithelial fate. This was nicely demonstrated by Kruse and colleagues who showed that limbal fibroblast conditioned media can induce the differentiation of hair follicle stem cells toward a corneal epithelial phenotype in vitro [12]. The specificity of these factors toward corneal cells is also highlighted by our results that a skin fibroblast conditioned media does not promote corneal epithelial cell growth in limbus to limbus scraped mice. In this study, human limbal fibroblast cultures were used given the difficulty obtaining and culturing murine limbal fibroblasts. However, future studies using murine fibroblasts may provide even more specific factors for the murine cornea.

While treatment with a limbal fibroblast conditioned media did not totally restore a corneal epithelial phenotype, it was found to be the predominant phenotype in the conditioned media cases. Anecdotally, we observed an increase in K12 expression in the cornea when going from 2 weeks to 3 weeks of treatment (data not shown). However, studies with longer duration and follow-up are necessary to determine if corneal epithelial cells will continue to become the predominant cell type. An observation worth noting is that in all cases, the corneal epithelial cells were arising from the limbus and were following a normal clonal growth pattern. In other words, based on the current study there is no evidence to suggest that limbal fibroblast conditioned media directly induced conjunctival epithelial cells in the central cornea to transdifferentiate into a corneal type epithelium.

In summary, this study has used a model of limbal stem cell deficiency to demonstrate the therapeutic benefit of limbal fibroblast conditioned media. The results further underscore the importance of limbal fibroblasts in the limbal niche. Given the relative absence of non-surgical treatments for partial limbal stem cell deficiency, such approaches may have potential clinical applications.
